# A Complex-Type *N*-Glycan-Specific Lectin Isolated from Green Alga *Halimeda borneensis Exhibits Potent* Anti-Influenza Virus Activity

**DOI:** 10.3390/ijms25084345

**Published:** 2024-04-15

**Authors:** Jinmin Mu, Makoto Hirayama, Kinjiro Morimoto, Kanji Hori

**Affiliations:** 1 Graduate School of Biosphere Science, Hiroshima University, Kagamiyama 1-4-4, Higashi-Hiroshima 739-8528, Japan; jinminmu1985@gmail.com (J.M.); hirayama@hiroshima-u.ac.jp (M.H.); 2 Graduate School of Integrated Sciences for Life, Hiroshima University, Kagamiyama 1-4-4, Higashi-Hiroshima 739-8528, Japan; 3 Faculty of Pharmacy, Yasuda Women’s University, Yasuhigashi 6-13-1, Asaminami-Ku, Hiroshima 731-0153, Japan; mori-k@yasuda-u.ac.jp

**Keywords:** lectin, green alga, *Halimeda borneensis*, complex *N*-glycan binding specificity, anti-influenza virus activity

## Abstract

Marine algal lectins specific for high-mannose *N*-glycans have attracted attention because they strongly inhibit the entry of enveloped viruses, including influenza viruses and SARS-CoV-2, into host cells by binding to high-mannose-type *N*-glycans on viral surfaces. Here, we report a novel anti-influenza virus lectin (named HBL40), specific for complex-type *N*-glycans, which was isolated from a marine green alga, *Halimeda borneensis*. The hemagglutination activity of HBL40 was inhibited with both complex-type *N*-glycan and *O*-glycan-linked glycoproteins but not with high-mannose-type *N*-glycan-linked glycoproteins or any of the monosaccharides examined. In the oligosaccharide-binding experiment using 26 pyridylaminated oligosaccharides, HBL40 only bound to complex-type *N*-glycans with bi- and triantennary-branched sugar chains. The sialylation, core fucosylation, and the increased number of branched antennae of the *N*-glycans lowered the binding activity with HBL40. Interestingly, the lectin potently inhibited the infection of influenza virus (A/H3N2/Udorn/72) into NCI-H292 cells at IC_50_ of 8.02 nM by binding to glycosylated viral hemagglutinin (K_D_ of 1.21 × 10^−6^ M). HBL40 consisted of two isolectins with slightly different molecular masses to each other that could be separated by reverse-phase HPLC. Both isolectins shared the same 16 *N*-terminal amino acid sequences. Thus, HBL40 could be useful as an antivirus lectin specific for complex-type *N*-glycans.

## 1. Introduction

Influenza is a typical human endemic and epidemic disease caused by infection with the influenza virus, yet the disease remains difficult to predict or prevent [1]. Influenza viruses infect target cells and propagate using two viral envelope glycoproteins with *N*-acetylneuraminic acid-binding properties, hemagglutinin (HA) and neuraminidase (NA) [1]. HA binds to terminal *N*-acetylneuraminic acid residues of receptors on infected cells, triggering virus internalization into the cell. NA, on the other hand, is responsible for cleaving *N*-acetylneuraminic acid residues on the virus and infected cell surface, allowing the proliferating virus to be released from the cell, and also aids virus attachment and entry into target cells [1,2]. The number of HAs on the viral surface is up to 10-fold more than NAs [1].

In contrast, the human body has an innate immune system that prevents viral infection, in which C-type lectins such as the mannan-binding protein (MBL) in serum and surfactant protein D (SP-D) on the surface of lung cells play a major role against influenza viruses [3]. MBL binds to sugar determinants of several microorganisms, including viruses, and can inhibit infection by complement activation and opsonization via the lectin pathway, followed by phagocytosis [3]. People who are genetically deficient in MBL or who have low-serum MBL are more susceptible to viral infections [4]. Native MBL isolated from serum and its recombinant form produced in *E. coli* were also shown to directly block the viral infection of host cells by binding to viral envelope proteins, HA and NA, and their activities were inhibited by mannose, EDTA, and the anti-human MBL antibody [3]. Thus, it is suggested that MBL inhibits viral infection by binding to mannose residues on the influenza virus envelope. Other C-type lectins, such as macrophage mannose receptor (MMR) and DC-specific intercellular adhesion molecule-3-grabbing non-integrin (DC-SIGN), also inhibit the influenza virus infection by binding to mannose residues on the viral envelope HA and NA [1].

HA and NA are glycoproteins with several *N*-glycosylation sites where high mannose(HM)-type, hybrid-type, and complex-type *N*-glycans are attached, depending on the glycosylation pathway in host cells [1,5,6,7]. The HA of influenza A has shown a trend toward increasing numbers of glycans in comparison between early A/Hong Kong/1/1968 (H3N2) and recent A/Singapore/INFIMH-16-0019/2016 (H3N2) isolates and between early A/South Carolina/1/1918(H1N1) and recent A/Brisbane/59/2007(H1N1) isolates [1]. As *N*-glycans affect the virulence and immunogenicity of influenza viruses [1], glycans can be a potential target for antiviral therapy. From this perspective, several anti-influenza virus lectins have been discovered to date from natural sources, many of which show an HM-type *N*-glycan-binding property as well as MBL and SP-D, including the lectins from a cyanobacterium, such as *Nostoc ellipsosporum* (cyanovirin-N or CV-N) [8], the red algae, *Kappaphycus alvarezii* (KAA-2) [9] and *Eucheuma serra* (ESA-2) [10], the green algae, *Boodlea coacta* (BCA) [11] and *Halimeda renschii* (HRL40) [12], the proteobacteria, *Pseudomonas fluorescens* Pf0-1 (PFL) [13], *P. mandelii* (PML) [13] and *P. taiwanensis* (PTL) [13], and the land plants, *Musa acuminata* (BanLec) [14], *Hippeastrum hybrid* (HHA) [15] and *Galanthus nivalis* (GNA) [15]. Otherwise, there are a few reports on the anti-influenza viral activity of land plant lectins, including an *N*-acetylglucosamine (GlcNAc)-specific lectin from *Urtica dioica* (UDA) [15,16], a high mannose type/complex-type *N*-glyan-binding lectin (NICTABA) from *Nicotiana tabacum* [16], and a mannose/glucose-specific lectin from *Lablab purpureus* (FRIL) [17]. FRIL was shown to inhibit the infection of influenza A viruses through binding to complex-type *N*-glycans on HA, unlike most anti-influenza virus lectins, which show activity through binding to HM-type *N*-glycans [17]. Regarding legume lectins, Barre et al. summarized the characteristics of antiviral leguminous lectins against pathogenic-enveloped viruses, including influenza viruses [18].

Many of the anti-influenza virus lectins with binding specificity to HM-type *N*-glycans belong to the OAAH homolog lectin family [19], including KAA-2, ESA-2, PFA, PML, and PTL, which have a common molecular structure and bind preferentially to the HM-type *N*-glycans with non-reducing α1-3Man on the D2 arm [9,10,13]. On the other hand, HRL40, despite having a binding preference for the branched mannoside structure, similar to the OAAH family members and showing potent antiviral activity, possesses a different molecular structure from the OAAH family members [12]. Given the relatively large number of species of marine green algae belonging to the genus *Halimeda*, the *Halimeda* algae may be worth exploring as a source of new antiviral lectins.

The present report deals with a novel anti-influenza virus lectin with complex-type *N*-glycan specificity, which was isolated from a calcareous green alga, *Halimeda borneensis*.

## 2. Results

### 2.1. Purification of Complex-Type N-glycan-Specific Lectin HBL40

**Figure 1:**
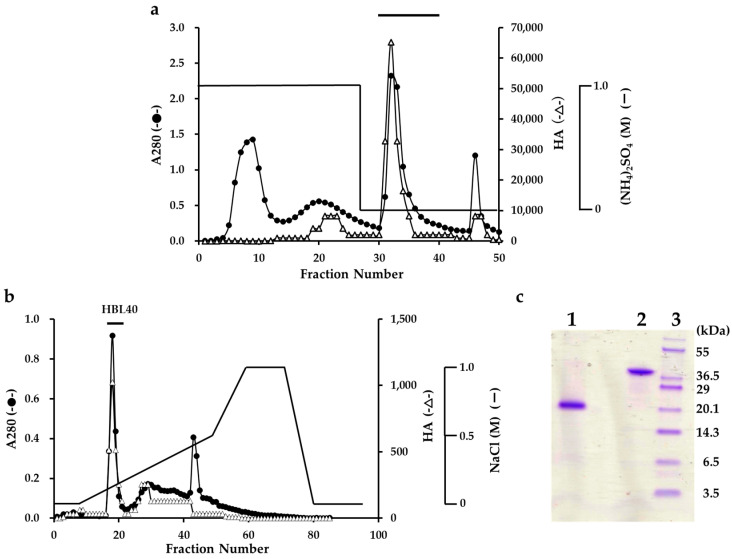
Purification of *H. borneensis* lectin (HBL 40). (**a**) Hydrophobic chromatography with step-wise elution on a HiPrep phenyl FF column (1.6 × 10 cm) of a precipitate with 75% saturation of ammonium sulfate (a salting out fraction). Eluates were fractionated 5 mL each. Each fraction was determined for absorbance at 280 nm (A_280_) (●) and for hemagglutination activity (∆). The active fractions denoted by a bar were collected. (**b**) Ion-exchange chromatography on a TSKgel DEAE-5PW column of the active fractions obtained by hydrophobic chromatography. The active peak, denoted by a bar in the figure, was recovered as a finally purified lectin (HBL40). (**c**) SDS-PAGE of a purified HBL40. The gel was stained with a CBB R-250 reagent. Lane 1, HBL40 with 2% 2-mercaptoethanol; lane 2, HBL40 without 2-mercaptoethanol; and lane 3, a molecular weight marker.

The lectin was extracted from an algal powder of *H. borneensis* with a 20 mM phosphate buffer containing 0.85% NaCl (PBS, pH 7.0), which was then precipitated with a 75% saturation of ammonium sulfate. In the hydrophobic chromatography of the salting-out fraction using a HiPrep Phenyl column, the lectin was adsorbed onto the column and then eluted with 20 mM PB (Figure 1a). The PB eluate gave two active peaks in the successive ion-exchange chromatography using a TSKgel DEAE-5PW column. The major active peak was eluted with about 0.2 M NaCl and recovered as a finally purified lectin fraction (Figure 1b).

The purified lectin showed a single protein band of about 40 kDa in non-reducing and 20 kDa in reducing sodium dodecyl sulfate-polyacrylamide gel electrophoresis (SDS-PAGE) (Figure 1c). The yield of the purified lectin, named HBL40, was 4.8 mg per 100 g of the frozen algal sample (Table 1).

### 2.2. Hemagglutination-Inhibition Test by Sugars and Glycoporteins

The hemagglutination activity of HBL40 was not inhibited by the monosaccharides examined but strongly inhibited by some glycoproteins bearing complex-type *N*-glycans (transferrin, porcine thyroglobulin (PTG), fetuin) and *O*-glycans (bovine submaxillary mucin (BSM)). The inhibition activities of the asialo-derivatives of *N*-glycan-linked glycoproteins were stronger than those of parent sialo-glycoproteins. On the other hand, yeast mannan, bearing HM type *N*-glycan, was not inhibitory. The inhibition profiles of the purified lectin HBL40 adequately resembled those of a salting-out fraction and a partially purified fraction (Table 2). From the hemagglutination-inhibition test, HBL40 was considered to have a preference binding affinity for complex-type *N*-glycans and was clearly distinct from the anti-influenza virus lectin (HRL40) from *H. renschii*, which had an affinity for HM-type *N*-glycans [12].

### 2.3. Binding Specificity to Oligosaccharides

**Figure 2:**
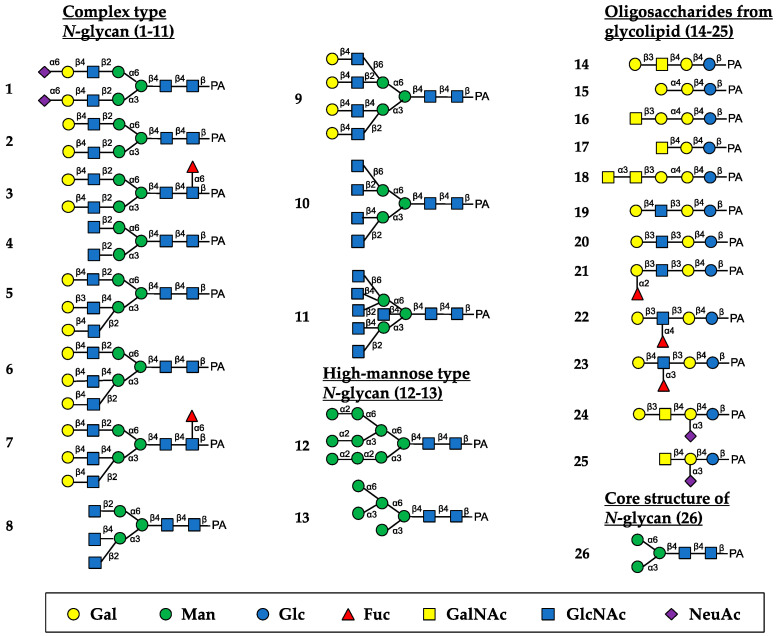
Schematic structures of PA-oligosaccharides examined for the oligosaccharide-binding assay of HBL40. The PA-oligosaccharides used in this study included complex-type *N*-glycans (1–11), HM-type *N*-glycans (12 and 13), oligosaccharides originating from glycolipids (14–25) and an *N*-glycan core pentasaccharide (26). The monosaccharide residues of Gal, Man, Glc, Fuc, GalNAc, GlcNAc, and NeuAc are represented as symbols shown in the black box.

The oligosaccharide-binding specificity of HBL40 was determined by a centrifugal ultrafiltration-HPLC method [20] using complex-type *N*-glycans (1–11, as numbered in Figure 2), HM-type *N*-glycans (12 and 13), oligosaccharides originating from glycolipids (14–25) and an *N*-glycan core pentasaccharide (26) (Figure 2). The binding activity for the examined pyridylaminated (PA)-oligosaccharides was represented as the ratio (%) of the amount of a bound PA-oligosaccharide to that of an added PA-oligosaccharide.

**Figure 3:**
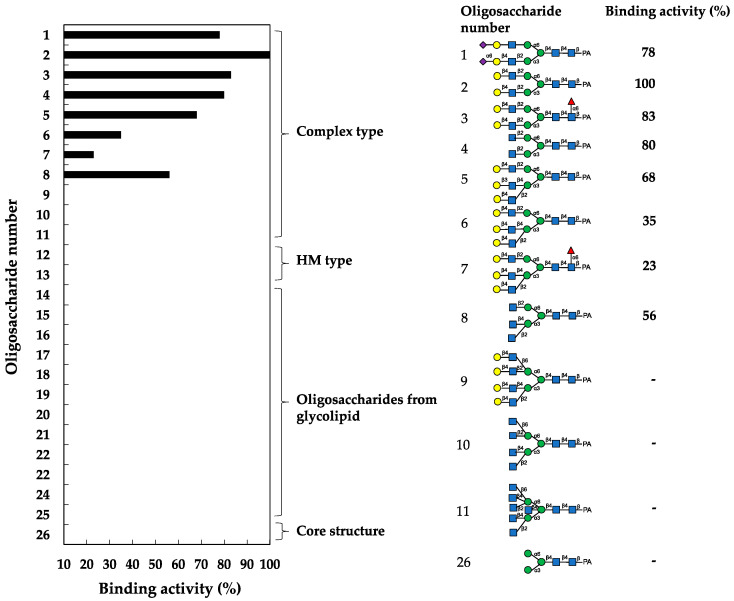
Binding activity of HBL40 to PA-oligosaccharides. The oligosaccharide-binding activity was examined using a centrifugal ultrafiltration-HPLC method [20]. The binding activity was expressed as a ratio (%) of the amount of bound PA-oligosaccharide [O_bound_] to that added [O_added_], where [O_bound_] was obtained by subtracting the amount of unbound PA-oligosaccharide [O_unbound_] from [O_added_]. [O_unbound_] was determined by reverse-phase HPLC, as described in the Section 4. The experiments were performed in triplicate for each PA-oligosaccharide, and the activity was obtained as the average value. The activities of less than 10% were cut off for their insignificance.

In the assay, HBL40 exclusively bound complex-type *N*-glycans (1–8) with bi- and triantennary-branched sugar chains and did not bind other oligosaccharides including tetra- and pentaantennary complex *N*-glycans (9–11), HM type *N*-glycans (12 and 13), oligosaccharides originating from glycolipids (14–25) and an *N*-glycan core pentasaccharide (26). Thus, the binding preference of HBL40 for complex-type *N*-glycans was dependent on the branched antenna structure. As shown in Figure 3, the highest binding activity was detected with an oligosaccharide 2 (asialo-biantennae) (binding activity, 100%), with a little less binding activity for oligosaccharides 3 (asialo-biantennae with core fucose) (83%), 4 (agalacto-biantennae) (80%) and 1 (sialo-biantennae) (78%), and moderate activity with oligosaccharides 5 (asialo-triantennae) (68%) and 8 (agalacto-triantennae) (56%), whereas no activity was observed with oligosaccharides 9 (asialo-tetraantennae), 10 (agalacto-tetraantennae), 11 (asialo-pentaantennae) and 26 (core structure). Thus, HBL40 preferentially recognized bi- and triantennary complex-type *N*-glycans, although it had no interaction with tetra- and pentaantennary ones. Among bi- and triantennary chains, sialylated and fucosylated derivatives had a tendency to lower the binding affinity of HBL40, as shown by the comparison of binding activities between oligosaccharides 1 and 2, oligosaccharides 2 and 3, and oligosaccharides 6 and 7, in Figure 3.

### 2.4. Inhibition of Virus Infection by HBL40

**Figure 4:**
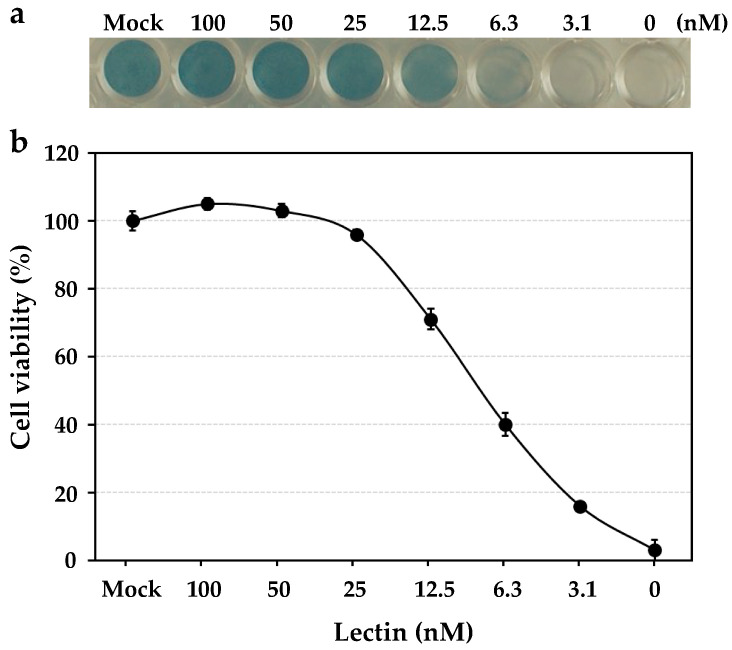
The dose-dependent anti-influenza virus activity of HBL40. NCI-H292 cells grown in a 48-well plate were infected with the influenza virus A/H3N2/Udorn/72 strain at a multiplicity of infection (moi) of 2.5 in the presence or absence of serially diluted lectin solutions. At 24 h post-infection (hpi), the infected cells were fixed with 80% acetone, following which they were stained with amide black (**a**). The experiments were performed in triplicate at each lectin concentration, and the cell viability was expressed as the mean +/− standard deviation (**b**), as described in the previous paper [12].

The anti-influenza virus activity was revealed by the cell viability of human NCI-H292 cells, which were infected by the influenza virus A/H3N2/Udorn/72 strain in the presence of the serially diluted solutions of HBL40. In the assay, HBL40 strongly prevented the entry of the influenza virus A/H3N2/Udorn/72 strain into NCI-H292 cells in a dose-dependent manner, as shown in Figure 4. The IC_50_ of HBL40 was 8.02 nM (Figure 4) by reading the point at which the curve crossed the 50% line, indicating that this lectin is also a promising antiviral agent.

### 2.5. Interaction between HBL40 and Envelope Hemagglutinin of Influenza Virus

**Figure 5:**
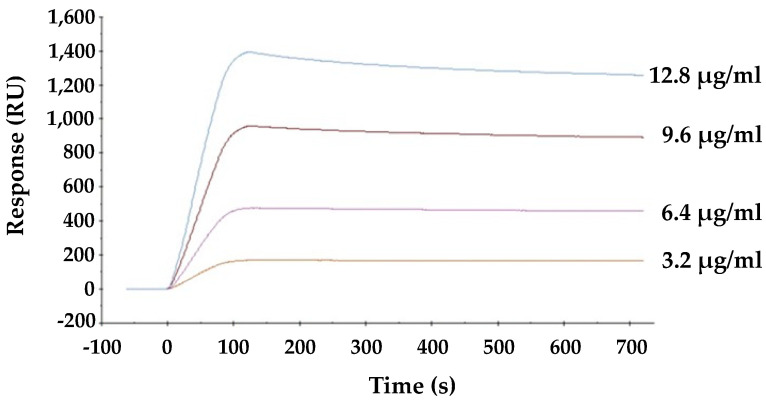
The interaction between HBL40 and influenza virus hemagglutinin was analyzed by SPR on a BIAcore X100 system (Cytiva, Tokyo, Japan).

The direct interaction between viral envelope hemagglutinin and HBL40 was analyzed by surface plasmon resonance (SPR). As shown in Figure 5, lectin is directly bound to the viral envelope hemagglutinin in a dose-dependent manner, and the affinity of HBL40 for the viral hemagglutinin was presented with the K_D_ value of 1.21 × 10^−6^ M (Table 3).

### 2.6. Molecular Structure of HBL40

**Figure 6:**
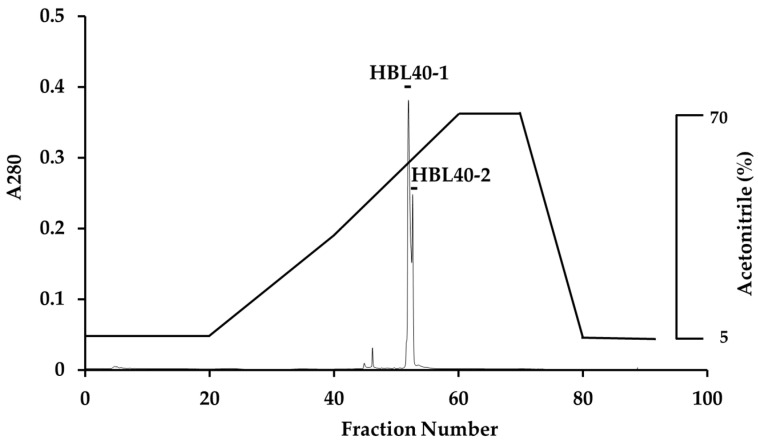
Reverse-phase HPLC of HBL40. Reverse-phase HPLC was conducted on a TSKgel ODS-80TM with a gradient elution of acetonitrile in 0.05% TFA. HBL40 was separated into two peaks, HBL40-1 and HBL40-2, denoted by a black bar.

**Figure 7:**
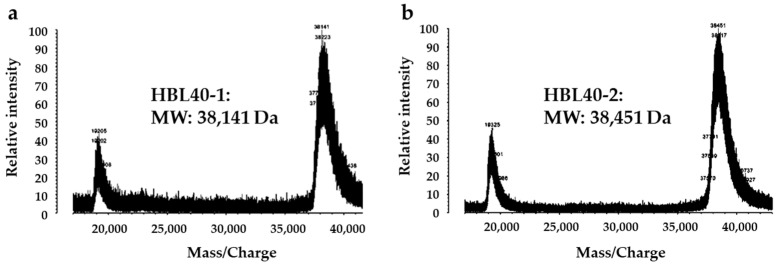
Matrix-assisted laser desorption/ionization time-of-flight mass spectrometry (MALDI-TOF-MS) of HBL40-1 (**a**) and HBL40-2 (**b**).

The relative molecular weight of the purified lectin HBL40 was estimated to be about 40 kDa in non-reducing and 20 kDa in reducing SDS-PAGE (Figure 1c), suggesting that HBL40 was a dimeric protein of a 20 kDa subunit. Meanwhile, HBL40 was further separated into two isoforms (HBL40-1 and HBL40-2) when it was subjected to reverse-phase HPLC on a TSKgel ODS-80TM column with a gradient elution of acetonitrile in 0.05% trifluoroacetic acid (TFA) (Figure 6). Matrix-assisted laser desorption/ionization time-of-flight mass spectrometry (MALDI-TOF-MS) revealed that the molecular weights of HBL40-1 and HBL40-2 were 38,141 Da and 38,451 Da, respectively (Figure 7).

The 16 *N*-terminal amino acid sequences of HBL40-1 and HBL40-2 were identical to ECGKNGFNCPSPLPAS, indicating that both are isolectins to each other. The significantly similar sequence with the *N*-terminal sequence of HBL40 was not found in databases.

## 3. Discussion

A new lectin, named HBL40, was purified from *Halimeda borneensis* by a combination of extraction, salting-out, and hydrophobic and ion-exchange chromatography in this order. The carbohydrate-binding specificity of HBL40 was clearly distinct from the lectin HRL40 purified from *H. renschii*, although both lectins were derived from the same genus of algae. In the hemagglutination-inhibition test, HBL40 showed an affinity for complex-type *N*-glycan-linked and *O*-glycan-linked glycoproteins (Table 2), while HRL40 showed an affinity for HM-type *N*-glycan-linked glycoproteins [12]. The oligosaccharide-binding experiment also demonstrated that HBL40 specifically recognized the complex type *N*-glycans with bi- and triantennary-branched chains and did not recognize those with tetra- and pentaantennary chains (Figure 3). The binding affinity was higher to the same extent with biantennary chains than with triantennary ones. Furthermore, Gal attached to the GlcNAc residues in the non-reducing terminal slightly increased the binding activity. The core (α1-6) Fuc and the reducing terminal NeuAc residues had the tendency to impair the affinity between HBL40 and the complex-type *N*-glycans. The slight change in the binding affinity with sialylated sugar chains fitted well with the hemagglutination-inhibition profile in which asialo-derivatives increased the inhibitory activity compared to sialo-glycoproteins.

HBL40 is the first complex-type *N*-glycan-specific lectin isolated from marine algae. On the other hand, there are a few plant lectins specific to complex-type *N*-glycans. For example, the *Phaseolus vulgaris*-erythroagglutinating lectin (PHA-E) recognizes complex-type *N*-glycans with bi- and triantennary-branched sugar chains, while it does not recognize those with tetra- and pentaantennary chains [21,22,23]. *Phaseolus vulgaris*-leucoagglutinating lectin (PHA-L) has binding preferences for tri- and tetraantennary complex *N*-glycans [21]. *Tulipa gesneriana* agglutinin (TGA) also shows a binding affinity for the bi- and triantennary complex *N*-glycans but not for tetraantennary types [24]. Thus, the complex *N*-glycan binding property of HBL40 resembled that of PHA-E and TGA with respect to the preference for the number of branched sugar chains. However, HBL40 differs from TGA in the effectiveness of the core (α1-6) Fuc residue, which decreases the binding affinity for HBL40 and increases the binding affinity for TGA. PHA-E, PHA-L, and TGA seem to have been reported thus far for their anti-influenza virus activity, although the anti-HIV-1 and anti-SARS-CoV-2 activities of PHA-E have been reported [25,26].

In this study, HBL40 inhibited the entry of the influenza virus A/H3N2/Udorn/72 strain into NCI-H292 cells at a low IC_50_ of 8.02 nM. This activity was comparable to the IC_50_ of high-mannose-type *N*-glycan-specific lectins, HRL40 (2.4 nM), PFL (10.5 nM), PML (10.5 nM), and PTL (9.5 nM), which were determined in a similar way with the same virus strain [12,13]. On the other hand, the affinity constant (K_D_) of HBL40 for the virus envelope hemagglutinin was 1.21 × 10^−6^ M and fairly higher than that of HRL40 (K_D_, 3.69 × 10^−11^ M). It is postulated that HBL40 inhibited the virus entry by binding to the complex *N*-glycans with bi- and triantennary sugar chains on HA, which differed from HRL40 exhibiting the inhibitory activity by binding to the HM-type *N*-glycans. The differences between HBL40 and HRL40 regarding the relationship between IC_50_ and K_D_ values may be attributed to several factors, which are listed as follows. (1) Both lectins recognize different glycan structures; HBL40 binds to complex-type *N*-glycans, while HRL40 binds to high mannose-type *N*-glycans. (2) IC_50_ and K_D_ were measured with different targets; IC_50_ was determined with the virus strain (A/H3N2/Udorn/72) while K_D_ was determined with envelope hemagglutinin preparation (Denka-Seken), including a mixture of A/California/7/09 (H1N1), A/Victoria/210/09 (H3N2) and B/Brisbane/60/08, which was produced in chicken eggs. (3) The number and location of *N*-glycosylation sites, the ratio of the types of *N*-glycan structures (complex, high mannose, hybrid), and the location of the attachment of each glycan structure on viral hemagglutinin differed between the virus strain and the envelope hemagglutinin preparation. The low IC_50_ value of HBL40 suggests that complex-type *N*-glycans on the hemagglutinin of the virus strain (A/H3N2/Udirn/72) may have a relatively high abundance ratio or be located near the receptor binding domain of the hemagglutinin molecule. However, it is unclear yet why HBL40 showed strong inhibitory activity comparable to that of HRL40 despite its extremely low affinity for viral hemmagglutinin compared to HRL40, along with the mechanism of inhibition. On the other hand, the complex-type *N*-glycan-binding lectin FRIL from the edible Lablab beans has been investigated in detail for its inhibition mechanism against viral infection [17]. FRIL is a typical legume lectin that has a 48% sequence identity to the well-known mannose-specific concanavalin A (Con A), but unlike ConA, FRIL binds to complex-type *N*-glycans on HA and can neutralize 11 typical human and avian influenza strains at low nanomolar concentrations [17]. This lectin exerts its antiviral effect as follows: FRIL first binds virions, and then the FRIL–virus complex is endocytosed into host cells, where the FRIL-bound virus is retained in the late endosome/lysosome and prevents nuclear entry until its ultimate degradation [17]. FRIL was also demonstrated to possess the activity in vivo by intranasal administration in mice [17].

HBL40 seems to have an advantage as a research tool or a therapeutic agent that strictly binds to complex-type *N*-glycans, in comparison with FRIL, which shows a broad binding property for both complex-type and high-mannose-type *N*-glycans besides the monosaccharides, mannose, and glucose [17]. On the HA of influenza viruses propagated through mammalian cells, complex type *N*-glycans are significant compared to the high mannose type. Therefore, it is possible that the lectins recognizing complex-type *N*-glycans block viral infection by binding to the glycans on HA. Thus, HBL40 could be a valuable tool for clinical research on influenza virus infection.

In this study, the anti-influenza activity of an algal lectin, HBL40, was demonstrated by an in vitro experiment using cultured human NCI-H292 cells and the influenza virus A/H3N2/Udorn/72. On the other hand, it is important to examine the in vivo inhibitory effect of the lectin because the local organization and environment of the receptors or other cell surface molecules may alter the binding specificity of the lectin [27]. Furthermore, the infections of influenza viruses are affected by heterologous polarized cells with virus receptors in the respiratory tract, which form an apical surface facing the external environments and a basal surface attached to the basement layer [28]. These reports suggest the presence of complex sites in the airway where lectins may act, as well as the importance of in vivo experiments for anti-influenza virus activity in future work.

## 4. Materials and Methods

### 4.1. Materials

The algal specimen of *H. borneensis* was collected on the coast of Harutahama in Yakushima, Kagoshima, in July 2012. The algal sample was kept at −30 °C until used. The species identification was performed based on the plastid elongation factor Tu (*tufA*) gene sequence, as shown in the Supplementary Information (Figure S1), besides morphological observations.

The HiPrep Phenyl FF column was purchased from Cytiva (Tokyo, Japan), and the TSKgel DEAE-5PW column was obtained from Tosoh Co. (Tokyo, Japan). d-Glucose (Glc), d-galactose (Gal), *N*-acetyl-d-galactosamine (GalNAc), transferrin, fetuin, bovine submaxillary mucin (BSM), and porcine thyroglobulin (PTG) were obtained from Sigma-Aldrich Co. (St. Louis, MO, USA). d-Mannose (Man), l-fucose (Fuc), *N*-acetyl-d-glucosamine (GlcNAc), *N*-acetyl-d-neuraminic acid (NeuAc), lactose, and yeast mannan were purchased from Nacalai Tesque Co. (Kyoto, Japan). d-Xylose (Xyl) and l-rhamnose (Rha) were purchased from FUJIFILM Wako Pure Chemical Co. (Osaka, Japan). The desialylated derivatives of transferrin, fetuin, BSM, and PTG were prepared by the hydrolysis of the parent sialoglycoproteins with 0.1 N HCl for 1 h at 80 °C. PA-oligosaccharides were purchased from Takara Bio (Tokyo, Japan). All other reagents used in this study were of the highest purity available. The influenza virus strain A/H3N2/Udorn/72 was supplied by Takemasa Sakaguchi (Graduate School of Biomedical & Health Sciences, Hiroshima University, Hiroshima, Japan), and the NCI-H292 cell (ATCC #CRL1848) was purchased from Culture Collections, Public Health England (London, UK).

### 4.2. Purification of Lectin HBL40 from H. borneensis

The frozen sample (100 g) of *H. borneensis* was thawed and ground in liquid nitrogen to a powder. To the powdered alga, 2 volumes (*v*/*w*) of PBS were added and stirred at 4 °C overnight. The mixture was centrifuged at 9000× *g* for 30 min at 4 °C. To the supernatant that was recovered, solid ammonium sulfate was added to attain a 70% saturation. The mixture was gently stirred for 30 min and kept at 4 °C overnight. The precipitates were recovered after centrifugation (9000× *g*, 30 min, 4 °C), dissolved in a small volume of PBS, and dialyzed thoroughly against the same buffer. The inner fraction was further centrifuged (9000× *g*, 30 min, 4 °C) to remove the insoluble components generated during dialysis, and the supernatant was recovered as a salting-out fraction. The salting-out fraction was adjusted to a 1 M solution with solid ammonium sulfate and applied to a HiPrep Phenyl FF column (1.6 cm × 10 cm), equilibrated with a 1 M ammonium sulfate in 20 mM PB. The column was thoroughly washed with the starting solution and then eluted with 20 mM of PB. The flow rate was 2 mL/min. Fractions of 5 mL were collected and measured for A_280_ with hemagglutination activity (HA). The active fractions eluted with 20 mM PB were pooled and concentrated by ultrafiltration (MWCO of 10 kDa). The concentrate was dialyzed against a 20 mM Tris-HCl buffer (pH 8.0) and applied to a TSKgel DEAE-5PW column (7.5 × 75 mm) equilibrated with the same buffer. The column was washed with the starting buffer and then eluted with a linear gradient of NaCl (0–1 M) in the buffer. Fractions of 1 mL were collected and measured for A_280_ and HA.

### 4.3. Determination of Protein Contents

The protein contents were determined by absorbance at 280 nm (A_280_) with the assumption that the protein solution of 1 mg/mL shows A_280_ of 1.0.

### 4.4. Sodium Dodecyl Sulfate–Polyacrylamide Gel Electrophoresis

Sodium dodecyl sulfate–polyacrylamide gel electrophoresis (SDS-PAGE) was carried out using 12% polyacrylamide gel, according to the method described by Schägger and Jagow [29]. After electrophoresis, the gel was stained with Coomassie brilliant blue R-250.

### 4.5. Hemagglutination Activity and Hemagglutination-Inhibition Test

Hemagglutination activity and the hemagglutination-inhibition test were performed with a 2% (*v*/*v*) suspension of trypsin-treated rabbit erythrocytes, as previously described [12]. The rabbit blood was purchased from the Hiroshima Animal Research Institute (Hiroshima, Japan). The following sugars and glycoproteins were used in this study: Glc, Man, Gal, GalNAc, GlcNAc, Fuc, Xyl, Rha, NeuAc, and lactose as sugars, and transferrin, asialo-transferrin, fetuin, asialo-fetuin, BSM, asialo-BSM, PTG, asialo-PTG, and yeast mannan as glycoproteins.

The hemagglutination-inhibition activity was given as the lowest inhibition concentration of sugar (mM) or glycoprotein (μg/mL), at which the complete inhibition of the hemagglutinaton activity of a lectin solution of titer 4 was achieved.

### 4.6. Oligosaccharide-Binding Specificity Analysis

The oligosaccharide-binding specificity was determined by a centrifugal ultrafiltration-HPLC method, as described by Hori et al. [20]. Briefly, 500 nM of lectin solution (90 µL) and 300 nM of PA-oligosaccharide (10 µL) (Takara Bio) were mixed in 50 mM Tris-HCl (pH 7.0) and incubated at room temperature for 60 min. The unbound PA-oligosaccharide [O_unbound_] was recovered in the filtrate by centrifugation (10,000× *g*, 30 s) with a centrifugal ultrafiltration device (PALL, Port Washington, NY, USA) (MWCO of 10 kDa). The filtrate (20 µL) was applied to a TSKgel ODS-80TM column (4.6 × 150 mm) (Tosoh Co.) and eluted with 15% methanol in a 0.1 M ammonium acetate buffer at a flow rate of 1 mL/min at 40 °C. The eluate was monitored for PA-oligosaccharide at an excitation wavelength of 320 nm and an emission wavelength of 400 nm. Meanwhile, 90 μL of 50 mM Tris-HCl (pH 7.0) without lectin was mixed with the same PA-oligosaccharide, and the mixture was centrifuged, as described above. The filtrate was obtained and used as a blank, which represented the number of added PA-oligosaccharides. The amount of bound PA-oligosaccharide [O_bound_] was obtained by the following formula: [O_bound_] = [O_added_] − [O_unbound_]. The binding activity was defined as a ratio of [O_bound_] to [O_added_] and denoted as % binding. The binding experiments were performed in triplicate per PA-oligosaccharide, and the activity was expressed as an average value.

### 4.7. Molecular Weight Determination

Prior to mass spectrometry analyses, the purified lectin (HBL40) was applied to a TSKgel ODS-80TM column (4.6 × 150 mm) equilibrated with 5% acetonitrile in 0.05% aqueous trifluoroacetic acid (TFA), and then the column was eluted by a gradient of 5–70% acetonitrile in 0.05% TFA. The eluates were monitored by A_280_. Two separated lectins (HBL40-1, HBL40-2) were collected for molecular weight determination.

Mass spectrometry analyses were carried out by matrix-assisted laser desorption/ionization time-of-flight mass spectrometry (MALDI-TOF-MS) with AXIMA-CFR plus (Shimadzu Corporation, Kyoto, Japan).

### 4.8. Determination of N-Terminal Amino Acid Sequences

The *N*-terminal amino acid sequencing was carried out for two lectins (HBL40-1 and HBL40-2), which were purified by reverse-phase HPLC on a TSKgel ODS-80TM column, using a Procise 492 HT protein sequencing system (Thermo Fisher Scientific, Waltham, MA, USA).

### 4.9. Anti-Influenza Virus Activity Assays

Anti-influenza virus activity was determined according to the method described previously [12,13]. Human NCI-H292 cells (ATCC CRL1848) grown in a 48-well plate were infected with influenza virus A/H3N2/Udorn/72 strain at a multiplicity of infection (moi) of 2.5. Various concentrations of the lectin were added simultaneously into the cell cultures. At 24 h post-infection (hpi), the infected cells were fixed with 80% acetone and stained with 0.5% amide black in 45% ethanol and 10% acetic acid. The stained plates were pictured with a grayscale. The color densities of the pictures were quantitated by densitometry with the NIH-ImageJ 1.48v software. The infected cell cultures, in the absence of lectin, exhibited severe cytopathic effects, and almost all cells on the wells were gone, with the percentage of cell viability shown as 0%. On the other hand, cells in the mock-infected cell cultures were intact, in which the percentage of cell viability was shown as 100%.

### 4.10. Surface Plasmon Resonance Analysis

The direct interaction between HBL40 and the influenza viral envelope glycoprotein hemagglutinin was analyzed by surface plasmon resonance (SPR) on a BIAcore X100 system (Cytiva, Tokyo, Japan), as previously described [10,11,12,13]. The influenza vaccine preparation (Denka-Seken), which contains a mixture of the hemagglutinin proteins of A/California/7/09 (H1N1), A/Victoria/210/09 (H3N2), and B/Brisbane/60/08, was immobilized as the viral hemagglutinin onto the sensor chip, which was activated with *N*-hydroxysuccinimide/*N*-ethyl-*N′*-dimethylaminopropyl carbodiimide. Various concentrations of lectin solution were used for binding experiments with a running buffer of HBS-N consisting of 10 mM 4-(2-hydroxyethyl)-1-piperazineethanesulfonic acid (HEPES), 150 mM NaCl (pH 7.4) at a flow rate of 30 μL/min. The contact time and dissociation time were performed as 120 s and 600 s, respectively. Kinetic parameters (k_a_, k_d_, K_A_, and K_D_) were calculated by fitting the data to the Langmuir model for 1:1 binding using the Biacore X100 evaluation software (Cytiva, Tokyo, Japan).

## 5. Conclusions

A lectin, named HBL40, was isolated from a green alga *H. borneensis;* HBL40 is a dimeric protein with a 20 kDa subunit that specifically binds complex-type *N*-glycans with bi- and triantennary-branched sugar chains and shows a potent anti-influenza virus activity through binding to the viral hemagglutinin. This lectin is expected to be a new research tool and therapeutic agent for preventing influenza infection.

## Figures and Tables

**Table 1:** Purification of a lectin HBL40 from *H. borneensis*.

Purification Step	Volume (mL)	Protein (mg/mL)	HA ^a^	THA ^b^	MAC ^c^ (μg/mL)
Extraction	228	6.0	2048	466,944	2.93
Salting-out	24	6.8	65,536	1,572,864	0.10
HP ^d^	60	1.0	8192	491,520	0.12
IE ^e^	2	0.4	2048	4096	0.21

^a^ HA, hemagglutination activity (titer); ^b^ THA, total hemagglutination activity (HA × volume); ^c^ MAC, minimum agglutination concentration, the protein concentration of the highest dilution showing positive hemagglutination; ^d^ HP, hydrophobic chromatography; ^e^ IE, ion-exchange chromatography; a 10 mL portion of an active fraction (60 mL) in HP was applied.

**Table 2:** Hemagglutination-inhibition tests of a salting-out fraction, a partially purified lectin fraction (HP), and a purified lectin HBL40 with sugar compounds.

Sugar and Glycoprotein	Salting-Out	HP ^a^	HBL40
Sugar (mM)			
Monosaccharides ^b^	>100	>100	>100
Disaccharide			
Lactose	>100	>100	>100
Glycoprotein (μg/mL)			
*N*-glycan-linked glycoprotein			
Complex type			
Transferrin	>1000	>1000	500
Asialo-transferrin	62.5	7.8	62.5
High-mannose type			
Yeast mannan	>1000	>1000	>1000
Complex and high-mannose types			
Porcine thyroglobulin (PTG)	62.5	31.3	31.3
Asialo-PTG	7.8	7.8	15.6
*N/O*-glycan-linked glycoprotein			
Fetuin	125	125	1000
Asialo-fetuin	7.8	7.8	125
*O*-glycan-linked glycoprotein			
Bovine submaxillary mucin (BSM)	7.8	7.8	62.5
Asialo-BSM	15.6	7.8	31.3

Values in the table represent the lowest concentration of sugars (mM) and glycoproteins (μg/mL). that completely inhibited the hemagglutination activity of a lectin solution of a titer 4. A 2% suspension of trypsin-treated rabbit blood cells was used for the hemagglutination-inhibition test. ^a^ HP, hydrophobic chromatography; ^b^ d-glucose (Glc), d-mannose (Man), d-galactose (Gal), *N*-acetyl-d-galactosamine (GalNAc), *N*-acetyl-d-glucosamine (GlcNAc), l-fucose (Fuc), d-xylose (Xyl), l-rhamnose (Rha), and *N*-acetyl-d-neuraminic acid (NeuAc).

**Table 3:** Binding kinetics of the interaction between HBL40 and influenza virus hemagglutinin.

	k_a_ (M^−1^s^−1^)	k_d_ (s^−1^)	K_A_ (M^−1^)	K_D_ (M)
HBL40	3.56 × 10^4^	4.33 × 10^−2^	8.3 × 10^7^	1.21 × 10^−6^

k_a_, association rate constant; k_d_, dissociation rate constant; K_A_, association constant; K_D_, dissociation constant.

## Data Availability

The data are contained within the article.

## Supplementary Materials

The following supporting information can be downloaded at: https://www.mdpi.com/article/10.3390/ijms25084345/s1. References [30,31,32,33,34,35,36,37,38] are cited in the Supplementary Materials.
