# Supplementary material for: A Complex-Type N-Glycan-Specific Lectin Isolated from Green Alga Halimeda borneensis Exhibits Potent Anti-Influenza Virus Activity
Source: Int J Mol Sci. 2024 Apr 15;25(8):4345. doi: 10.3390/ijms25084345 (PMC11050134; doi:10.3390/ijms25084345)
Supplement: Supplementary file 1 [file ijms-25-04345-s001.zip › ijms-2947223-supplementary.pdf]

Supplementary Information

A Complex-Type *N*-Glycan-Specific Lectin Isolated from Green Alga *Halimeda borneensis Exhibits Potent* Anti-Influenza Virus Activity

Jinmin Mu [1], Makoto Hirayama [1,2], Kinjiro Morimoto [3] and Kanji Hori [1,2]*

1 Graduate School of Biosphere Science, Hiroshima University, Kagamiyama1-4-4, Higashi-Hiroshima 739-8528, Japan; jinminmu1985@gmail.com (J.M); hirayama@hiroshima-u.ac.jp (M.H.); kanhori@hiroshima-u.ac.jp (K.H.)
2 Graduate School of Integrated Sciences for Life, Hiroshima University, Kagamiyama 1-4-4, Hi-gashi-Hiroshima 739-8528, Japan; hirayama@hiroshima-u.ac.jp (M.H.); kanhori@hiroshima-u.ac.jp (K.H.)
3 Faculty of Pharmacy, Yasuda Women's University, Yasuhigashi 6-13-1, Asaminami-Ku, Hiroshima 731-0153, Japan; mori-k@yasuda-u.ac.jp (K.M.)
* Correspondence: kanhori@hiroshima-u.ac.jp; Tel.: +81-82-424-7931

Table of contents:

- Materials and Methods pp. S2
- Results pp. S2
- References pp. S2-S3
- Supplementary Figure S1 pp. S4

S1. Materials and Methods

S1.1. Algal Specimens

Six specimens of *Halimeda* species (No. 1-5 and 15 in Figure S1) were collected on the coast of Harutahama in Yakushima Island, Kagoshima, in July 2007 (No. 1 and 2), 2011 (No. 3), 2012 (No. 4 and 5), and 2016 (No. 15). Six specimens (No. 6-11) in Ishigakijima Island, Okinawa, in October 2015, and three specimens (No. 12-14) were collected in Okinawa, in September 2015. Algal samples were kept at -30°C until used.

S1.2. DNA Extraction and Polymerase Chain Reaction (PCR)

The frozen algal tissues were thawed and ground in liquid nitrogen to a powder. Approximately 0.1 g of each powdered sample was transferred into 1.5-mL microtube, and subjected to DNA extraction using a MagExtractor - Plant Genome kit (TOYOBO, Osaka, Japan), according to the manufacturer's instruction. PCR amplification of the plastid *tufA* gene was carried out using a KOD FX Neo DNA polymerase (TOYOBO) in a total volume of 50 μL containing 25 μL of 2×PCR buffer for KOD FX, 10 μL of 2 mM dNTPs, 3 μL each of 10 μM of forward and reverse primers, 11 μL of DNA extract and 1 μL of KOD FX Neo (1.0 U/μL). The primers used for the PCR were as follows: tufA-A1 (5′-ATGRCWCGHGMAAAATTTSAACG-3′) and tufA-R (5′-CCTTCNCGAATMGCRAAWCGC-3′) [S1]. The touch down PCR was carried out as described by Kojima *et al.* [S2].

S1.3. DNA Sequencing

PCR products were monitored on a 1.0% agarose gel and the corresponding DNA bands were purified by a NucleoSpin Gel and PCR Clean-Up kit (Takara Bio, Shiga, Japan) following the manufacturer's instruction. After purification, the PCR products were sequenced by using a BigDye Terminator Cycle Sequencing kit ver. 3.1 (Thermo Fisher Scientific, IL, USA) with an ABI 3130xl genetic analyzer (Thermo Fisher Scientific). For the molecular phylogenetic analyses, 11 published sequences of Japanese *Halimeda* species and 2 outgroup ones were downloaded from GeneBank [S2] and added to the sequence alignments. The phylogenetic tree was obtained using maximum likelihood (ML) analyses implemented in the PhyML program (v3.1/3.0 aLRT) via Phylogeny.fr [S3-S9].

S2. Results

S2.1. Phylogenetic Analysis for Halimeda *specimens*

The phylogenetic analysis using a total of 28 *tufA* sequences of 15 *Halimeda* samples and 13 references (eleven *Halimeda* species and two outgroup ones) suggested that the fifteen samples were identified into 8 species: *H. macroloba* (No. 1), *H. kanaloana* (No. 2), *H. renschii* (No. 3, 4 and 15) and *H. borneensis* (No. 5, 8, 9, 11 and 13), *H. opuntia* (No. 6 and 7), *H. minima* (No. 10), *H. velasquezii* (No. 12) and *H. discoidea* (No. 14), as shown in Figure S1. The algal specimen No. 5 identified as *Halimeda borneensis* was used for further analysis in this study.

S3. References

S1. Famà, P.; Wysor, B.; Kooistra, WHCF.; Zuccarello, GC. Molecular phylogeny of the genus *Caulerpa* (Caulerpales, Chlorphyta) inferred from chloroplast *tufA* gene. *J. Phycol.* **2002**, 38, 1040-1050.

S2. Kojima, R.; Hanyuda, T.; Kawai, H. Taxonomic re-examination of Japanese *Halimeda* species using genetic markers, and proposal of a new species *Halimeda ryukyuensis* (Bryopsidales, Chlorophyta). *Phycol. Res.* **2015**, 63, 178-188.

S3. Castresana J. Selection of conserved blocks from multiple alignments for their use in phylogenetic analysis. *Mol. Biol. Evol.* **2000**, 17, 540-552.

S4. Guindon S.; Gascuel O. A simple, fast, and accurate algorithm to estimate large phylogenies by maximum likelihood. *Syst. Biol.* **2003**, 52, 696-704.

S5. Edgar RC. MUSCLE: multiple sequence alignment with high accuracy and high throughput. *Nucleic Acids Res*. **2004**, 32, 1792-1797.

S6. Anisimova M.; Gascuel O. Approximate likelihood ratio test for branchs: A fast, accurate and powerful alternative. *Syst. Biol*. **2006**, 55, 539-552.

S7. Chevenet F.; Brun C.; Banuls AL.; Jacq B.; Chisten R. TreeDyn: towards dynamic graphics and annotations for analyses of trees. *BMC Bioinformatics* **2006**, 7, 439

S8. Dereeper, A.; Guignon, V.; Blanc, G.; Audic, S.; Buffet, S.; Chevenet, F.; Dufayard, JF.; Guindon, S.; Lefort, V.; M. Lescot, M.; Claverie, JM.; Gascuel, O. Phylogeny.fr: robust phylogenetic analysis for the non-specialist. *Nucleic Aisds Res.* **2008**, 1, w465–w469.

S9. Dereeper, A.; Audic, S.; Claverie, JM.; Blanc, G. BLAST-EXPLORER helps you building datasets for phylogenetic analysis. *BMC Evol. Biol.* **2010**, 10, 8.

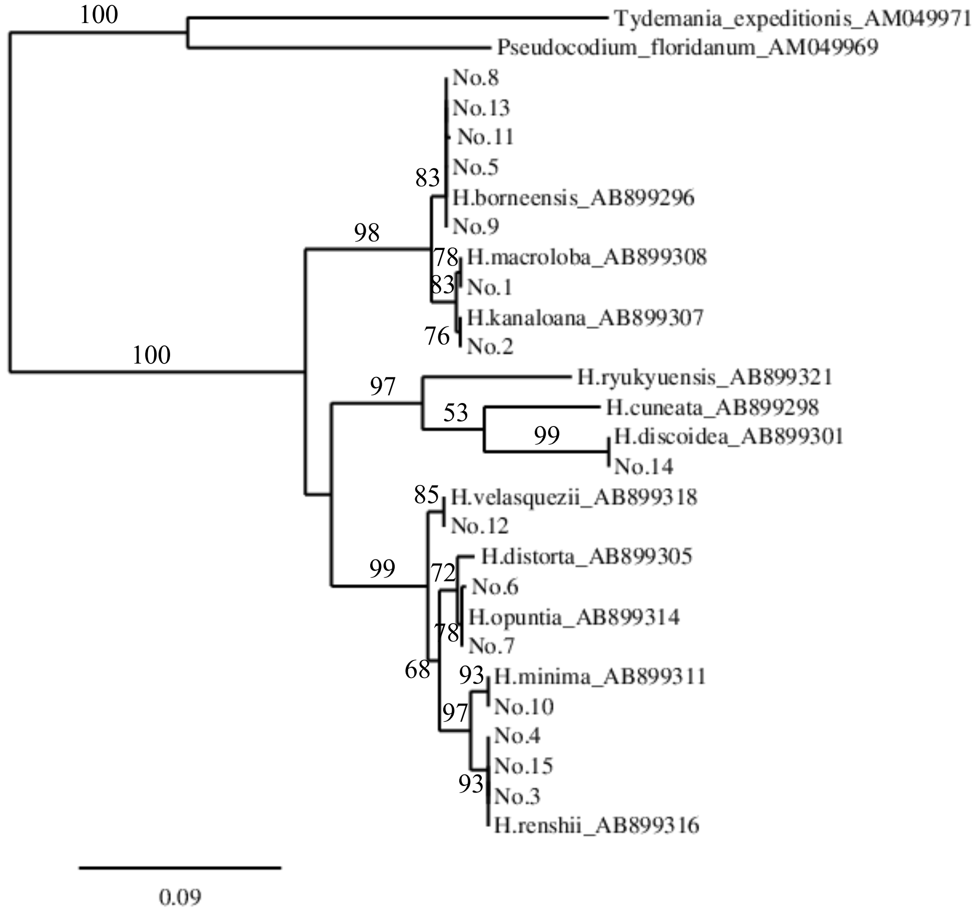

Figure S1. Phylogenetic analysis of Japanese *Halimeda* species based on DNA sequences of the gene *tufA*. Scale bar means 0.09 substitutions per site. The phylogenetic tree was constructed using the maximum likelihood method and the bootstrap values in percentage of more than 50 are presented in the tree.
